# Analysis of the Interrelation and Seasonal Variation Characteristics of the Spatial Niche of Dominant Fishery Species—A Case Study of the East China Sea

**DOI:** 10.3390/biology13090751

**Published:** 2024-09-23

**Authors:** Yong Liu, Jiahua Cheng

**Affiliations:** Key Laboratory of East China Sea Fishery Resources Exploitation, Ministry of Agriculture and Rural Affairs, East China Sea Fisheries Research Institute, Chinese Academy of Fishery Sciences, Shanghai 200090, China

**Keywords:** East China Sea, fishery resources, habitat suitability index, overlap relationship, seasonal change

## Abstract

**Simple Summary:**

This study examines the spatial distribution patterns of key fishery resources in the East China Sea across four seasons. Utilizing a habitat suitability index model, we identified the primary environmental factors influencing these patterns. Our results indicate that water temperature is a crucial factor for hairtail, while salinity and water depth are significant for small yellow croaker and Bombay duck, respectively. We also assessed the ecological niche overlap among different species and seasons, finding that predator-prey interactions primarily drive spatial habitat overlap. During summer and autumn, multiple species show increased overlap due to synchronized life cycles. An overlap index analysis demonstrated that species overlap rises from spring to winter, peaking in winter due to overwintering behavior and reduced food competition. These findings provide insights into species interactions and inform effective fishery resource management.

**Abstract:**

The spatial niche has garnered significant attention in ecological research, particularly regarding species distribution patterns. The East China Sea, known for its favorable natural conditions and abundant fishery resources, exhibits diverse spatial distribution patterns among species, shaped by their seasonal physiological needs. This study utilized a habitat suitability index model to explore the spatial distribution patterns of key fishery resources in the East China Sea across four seasons and their interactions. Two methodologies were employed to identify key environmental factors and assess the ecological niche overlap among different species and seasons. Results indicated that the initial method identified water temperature as the critical factor for hairtail, while the subsequent method emphasized water temperature and salinity for hairtail, salinity for small yellow croaker, and water depth for Bombay duck. The main spatial habitat overlap was observed between paired species, likely driven by predator-prey interactions. During summer and autumn, increased overlap among multiple species was primarily influenced by synchronized life cycles. An overlap index formula quantified the seasonal species overlap, showing an increase from spring to winter, reflecting changes in convergent habitat preferences. The peak overlap occurred in winter, driven by overwintering, reduced food competition, and enhanced coexistence potential, while the lowest overlap was noted in spring as overwintering ended and predation and competition intensified.

## 1. Introduction

The ecological niche, also referred to as the small environment, eco-zone, eco-habitat, or eco-niche, describes the alignment between species and specific environmental conditions [1]. This alignment includes the species’ spatial and temporal positions, functional relationships, roles within associated populations [2], and its status and functions within biological communities or ecosystems [3]. The concept of the ecological niche, an abstract, and broad-ranging idea, can be generally classified into spatial or habitat ecological niches, nutritional ecological niches, and multi-dimensional or hyper-volume ecological niches [4,5].

Understanding the location and role of species in biological communities or ecosystems based on their unique spatial distribution is termed the spatial or habitat ecological niche. Ecological biogeography primarily focuses on the ecological niche, particularly species’ spatial or habitat ecological niches. Species distribution and its temporal dynamics are influenced by species attributes, environmental changes, and their interactions [6]. The Species Distribution Model (SDM) predicts species distribution based on environmental variables determined by geographical and climatic conditions, utilizing observed species distribution data to simulate the actual ecological niche of species, distinguishing it from the fundamental niche where species can potentially survive [7]. Due to the ease and speed of implementing SDM with extensive data, research content has expanded significantly. Examples include the use of climate data and global species distribution maps to characterize species’ ecological niches [8] and studies on the distinct habitat ecological niches of four species of Trichiurus hairtail in the Northwest Pacific [9]. These related research topics are actively pursued across various fields, and the SDM approach has gained widespread application [10,11].

The habitat suitability index (HSI) is a scoring model that integrates multiple variables indicating habitat suitability into a composite index to predict the habitat’s capacity to support a species, and infer potential species distributions or relative abundance [12]. It is a widely used tool in biogeographic and niche-based ecological studies and is considered part of the broader SDM framework [13,14]. In recent years, there has been a surge in research utilizing niche-correlated models to explore the patterns and processes underlying species distributions [15]. This research encompasses diverse topics, including the relationship between the annual suitable habitat area and species abundance [16], evidence of population shifts northward in response to global changes by analyzing temporal changes in suitable habitats [16] and the synchronized response of spatial habitat distribution patterns to El Niño events of varying intensities [17]. Numerous studies have demonstrated that research outcomes based on the HSI model are relatively reliable [12,13,14,15,16,17].

The East China Sea boasts superior natural conditions and abundant fishery resources, accounting for nearly half of the nation’s ocean fishing output [18]. This region is rich in species with complex interrelationships. However, relatively little research has been conducted on the ecological niches of these species, and even less on the relationships between these niches. The fishery resources in the East China Sea exhibit dynamic changes across four distinct seasons, corresponding to the various life cycle stages of different fishery species, each with specific physiological requirements. For instance, during reproductive migration, species seek suitable water bodies that support egg laying and meet the survival needs of newly born individuals. Similarly, during foraging or overwintering migration, species may aggregate due to shared needs or maintain a certain distance due to competition or predation [19]. These life cycle processes create unique ecological spaces with distinct characteristics. Traditional fishery resources in the East China Sea include hairtail(*Trichiurus japonicus*), small yellow croaker(*Larimichthys polyactis*), and silvery pomfret(*Pampus argenteus*), among other economically important species. These species play a dual role as primary contributors to fishery production and essential components of the East China Sea’s marine ecosystem. Understanding the ecological niches of key species in the East China Sea provides crucial theoretical support for their sustainable utilization and conservation.

This study aims to utilize the HSI model to analyze the seasonal variations in the spatial distribution of optimal habitats for key fishery resource species in the East China Sea. Specifically, it seeks to identify the key environmental factors influencing species distribution, determine species groups with closely related spatial distribution patterns, explore the interrelationships among species within these groups, and observe the seasonal patterns of spatial overlap among species. The objective is to improve our understanding of the ecological roles and functions of these important species within the East China Sea’s fishery resources ecosystem and to provide technical support for the conservation and sustainable utilization of these vital fishery resources.

## 2. Materials and Methods

### 2.1. Data Source

#### 2.1.1. Resource Survey Data

**Figure 1:**
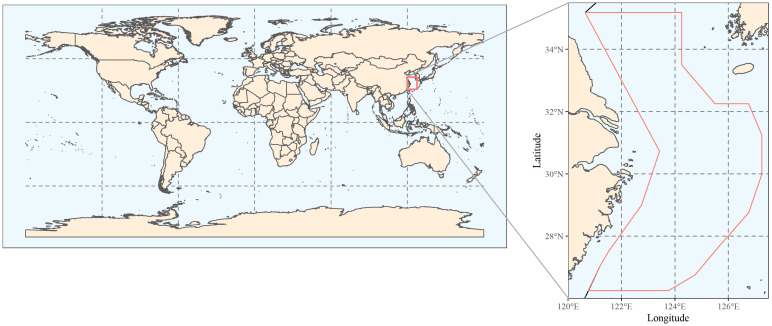
Schematic diagram of the study area.

Data were collected from four large-scale fixed-station surveys of fishery resources in the East China Sea conducted in May (spring), August (summer), and November (autumn) of 2014 and January (winter) of 2015. The survey vessel was a double trawler equipped with net gear measuring 100 meshes by 4 m, with a mesh size of 2.5 cm, and an average towing speed of 2 miles per hour. The survey area extended from 27°00′ to 34°00′ N and 122°00′ to 127°00′ E, with sampling stations set at 30′ intervals in a grid pattern, as shown in Figure 1.

#### 2.1.2. Environment Data

At each survey station, after retrieving the trawl net, a Conductivity-Temperature-Depth (CTD) profilerwas deployed to the sea floor for a brief pause before retrieval, collecting hydrological data including water temperature, salinity, and depth at each station. Since the survey vessel was a bottom trawler targeting bottom dwelling marine species, the environmental factors influencing species distribution were assumed to be related to the hydrological conditions at the sea bottom. Therefore, all hydrological data analyzed in this study pertain to bottom data. Due to various factors, CTD environmental data were not obtained from all stations. Missing environmental data were supplemented using model data from the Copernicus Marine Environment Monitoring Service [20]. Specifically, environmental data were not collected from waters south of 28° N during both spring (May 2014) and autumn (November 2014).

### 2.2. Analysis Methods

#### 2.2.1. Screening of Major Fish Species by Season

Utilizing survey data, the combined weight of each species and the total catch weight across all survey sites for each season were calculated. The biomass proportion for each species was determined by dividing its total weight by the overall catch weight. Species with biomass proportions exceeding 5% in all seasons were selected for analysis in this study. The screening results are presented in Table 1. The selected species accounted for the majority of the total biomass in each season, with the lowest cumulative ratio being 49.5% in autumn, and the highest ratio reaching 70.8% in summer. Therefore, the selected species represent the main fishery resources of the East China Sea, and studying their habitat characteristics is crucial for understanding the primary distribution patterns and informing the management of fishery resources in this region.

#### 2.2.2. Index Calculation Method

##### Boosted Regression Trees

The boosted regression tree (BRT) model, which combines regression tree and boosting techniques is a robust statistical model well-suited for data fitting. It operates through a series of iterative training rounds, where each iteration refines the model based on the results of the previous round, ultimately creating a powerful predictive tool [21]. The BRT model is particularly effective for addressing regression and classification problems, managing non-linear relationships and navigating high-dimensional data spaces.

In this study, the BRT model is used to evaluate the significance of various environmental factors on the target index. This process involves assigning weights to these factors, which are then used to calculate the HSI. The BRT model was implemented using the dismo (1.3-3) package in the R programming language [22].

##### Suitability Index

The suitability index (SI) in this study was calculated using the fishing yield (g/h), which was fitted using the LOESS smoothing function to examine the relationship between environmental variables and fishing yield [23]. The SI value was then determined based on the predicted values of the smoothed model. The formula is as follows:

(1) $$SI=\frac{\hat{Y}-{\hat{Y}}_{min}}{{\hat{Y}}_{max}-{\hat{Y}}_{min}}$$

In the formula, $\hat{Y}$ is the unit fishing yield after smoothing regression, while ${\hat{Y}}_{max}$ and ${\hat{Y}}_{min}$ are the maximum and minimum values of the prediction values, respectively. The SI value rranges from 0 to 1; values closer to 1 indicate a higher SI, and values closer to 0 indicate a lower SI.

##### Habitat Suitability Index

The HSI is calculated by synthesizing multiple SIs. Based on the results of the BRT model, weights were assigned to different factors, leading to two HSI calculation methods used in this study: the arithmetic mean model (AMM) [24] and the geometric mean model (GMM) [25]. The formulas for calculation are as follows:

(2) $$HSI_{AMM}=\frac{1}{\sum_{i=1}^{n}w_{i}}\times \sum_{i=1}^{n}SI_{i}w_{i}$$

(3) $$HSI_{GMM}=(\prod_{i=1}^{n}SI_{i}^{w_{i}}{)}^{\frac{1}{\sum_{i=1}^{n}w_{i}}}$$

In these formulas, HSI is the habitat suitability index, $SI_{i}$ is the *SI* value of environmental variable i, $w_{i}$ is the weight assigned to environmental variable i through the BRT model, and n is 3, referring to the three environmental factors—temperature, salinity and water depth—selected in this study.

The calculation of the HSI for each species across different seasons involves using cross-validation to assess the predictive capabilities of the AMM and GMM models. A random selection of 80% of the data is used for model development, while the remaining 20% is reserved for model accuracy verification. This process is repeated 100 times [26]. During each iteration of cross-validation, a linear regression analysis is performed between the predicted HSI value and the observed value (the SI index derived directly from the catch data per unit time). The similarity between these two values is evaluated by establishing a linear relationship between the predicted and observed values from the test set. The Akaike Information Criterion (AIC) is used to assess the performance of the HSI model, determining the optimal model [27].

To visualize the spatial distribution of the HSI for each fish species, the geostatistical Kriging model was used to interpolate HSI distribution data based on the HSI values calculated at each site. The interpolation results occasionally included negative values, although the HSI is inherently non-negative. When plotting the HSI spatial distribution map, the interpolated HSI values were classified into five categories: values not exceeding zero were grouped into one category, and the remaining data were divided into four categories using equal intervals from 0 to 1. Different colors were assigned to these five categories to aid in visualization.

##### Overlap Index

To comprehensively analyze and compare the spatial overlap of species across different seasons, this study has devised an overlap index. The most suitable areas were categorized based on the number of overlapping species, including non-overlapping single-species optimal areas, pairwise overlapping optimal areas, and multi-species overlapping optimal areas (including 3 or more species). The ecological niche overlap’s importance, which correlates with the number of overlapping species, was weighted differently to calculate an overlap index ($I_{Overlap}$) for each season by multiplying the corresponding area. The calculation formula is as follows:

(4) $$I_{Overlap}=\sum_{i=1}^{n}w_{i}\times A_{i}\;\;\;,i=1\ldots n$$

In this formula, $w_{i}$ represents the weight value assigned based on the overlap level, with values of {−0.5, 1, 2} assigned for the three levels in this study. n refers to the number of overlap levels, which is 3 in this study. $A_{i}$ represents the percentage of the area corresponding to each overlap level relative to the total area of all levels.

### 2.3. The Software Used

The data processing and chart drawing in this study were conducted using R software (version 4.0.1) [28]. The main packages used for data processing and arrangement were RODBC (1.3–17) [29] and readxl (1.3.1) [30]. For drawing, the primary packages included mapdata (2.3.0) [31], maps (3.3.0) [32], mapplots (1.5.1) [33], and ggplot2 (3.3.6) [34]. The UpSetR (1.4.0) [35] package was utilized to illustrate the spatial distribution relationships among different fish species.

## 3. Results

### 3.1. Relative Importance of Environmental Factors by Season for Each Fish Species

**Figure 2:**
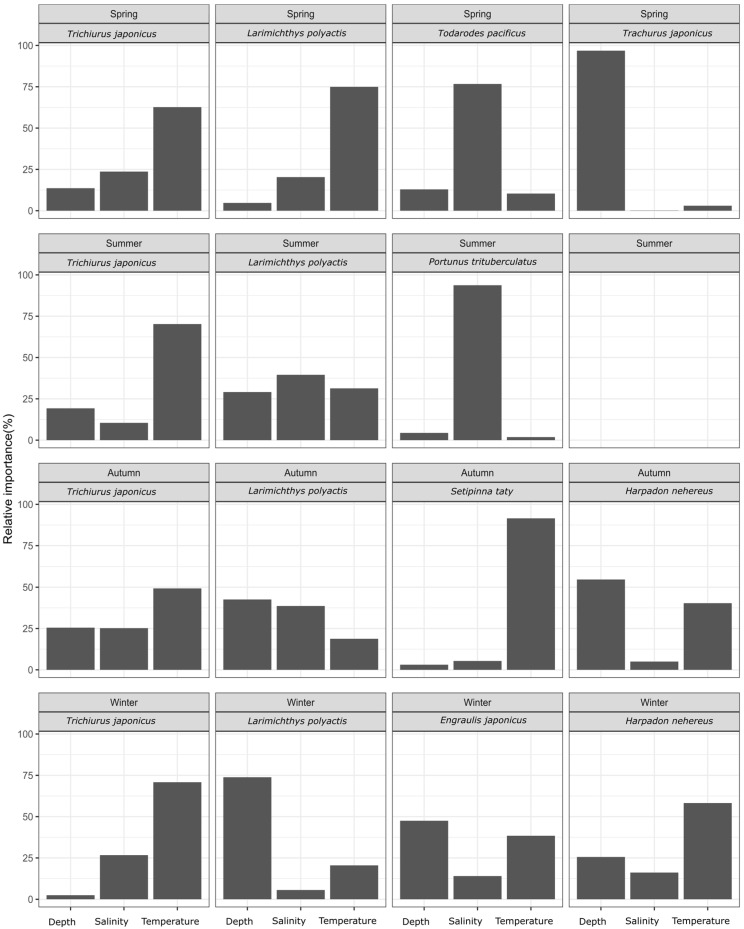
The relative importance of environmental factors for each fish species by season.

Based on the BRT model, the relative importance of temperature, salinity, and depth on the biomass of each dominant species was calculated, as shown in Figure 2. The analysis indicated that temperature was consistently the key factor affecting hairtail biomass, across all four seasons. For the small yellow croaker, key factors varied by season: temperature in spring and depth in winter, with no major factors identified in summer and autumn. Salinity was an important environmental factor for swimming crab in summer and the Pacific squid in spring. In autumn, temperature was the key factor for half-fin anchovy, while depth was crucial for the Japanese jack mackerel in spring. Neither Bombay duck nor anchovy had any predominant influencing factor in any season; however, Bombay duck in autumn and anchovy in winter were relatively more influenced by temperature and depth, respectively. Additionally, a stronger influence of temperature on Bombay duck was observed in autumn.

### 3.2. Smoothed Fitting Results of Environmental Factor SI

#### 3.2.1. Differences in the Environmental Suitability Selection among Fish Species in Each Season

**Figure 3:**
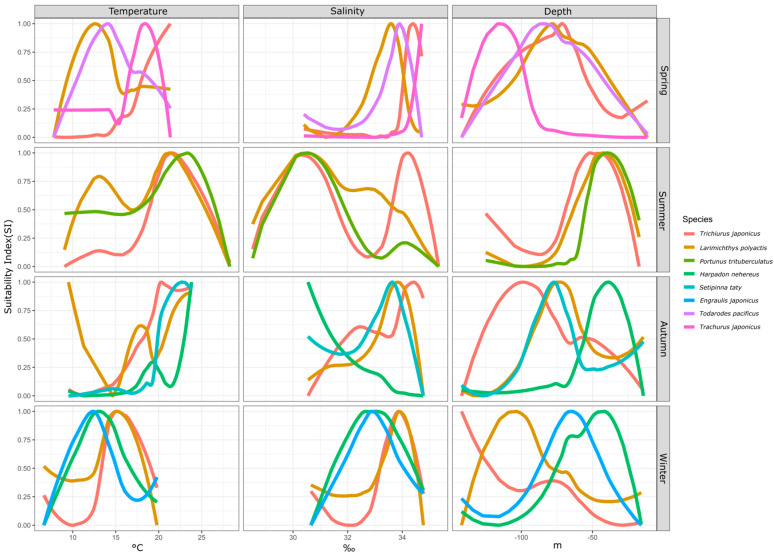
Interspecies differences in environmental SI by season.

Fish species exhibit suitable ranges for environmental factors in different seasons. By examining the suitability differences of specific environmental factors for fish species within a season, we can analyze the sources of ecological niche variations. This study compared the suitable ranges of temperature, salinity, and water depth among coexisting fish species across four seasons, as shown in Figure 3.

In spring, four fish species had a biomass proportion exceeding 5%: hairtail, small yellow croaker, Pacific squid, and Japanese jack mackerel. In terms of temperature suitability, small yellow croaker and Pacific squid had similar ranges, whereas hairtail and Japanese jack mackerel were more alike, with the latter group favoring higher average temperatures. The order of preferred temperature was hairtail > Japanese jack mackerel > Pacific squid > small yellow croaker. For salinity suitability, the four species were relatively close, with the order being Japanese jack mackerel > hairtail > Pacific squid > small yellow croaker. Regarding water depth suitability, Japanese jack mackerel preferred deeper waters, while hairtail, small yellow croaker, and Pacific squid showed similar depth preferences.

In summer, three fish species had a biomass proportion exceeding 5%: hairtail, small yellow croaker, and swimming crab. For temperature suitability, all three species exhibited a consistent optimal range of 20–25 °C, while small yellow croaker and swimming crab also had a secondary range of 10–15 °C. Regarding salinity suitability, the three species shared a common range of 30–31‰, with small yellow croaker and hairtail displaying distinct ranges around 33‰ and 34‰, respectively. In terms of water depth suitability, all three species consistently had a suitable range of around 50 m.

In autumn, four fish species had a biomass proportion exceeding 5%: hairtail, small yellow croaker, half-fin anchovy, and Bombay duck. For temperature suitability, the small yellow croaker had three optimal ranges around 10 °C, 18 °C, and 23 °C. The other three species favored temperatures above 20 °C, in the order of Bombay duck > half-fin anchovy > hairtail, with hairtail showing the broadest range. Concerning salinity suitability, except for the Bombay duck, which preferred lower salinity, the other three species had relatively high and similar salinity preferences. For water depth suitability, the range for small yellow croakers overlapped with that of half-fin anchovy, while hairtail preferred the deepest range and Bombay duck the shallowest.

In winter, four fish species had a biomass proportion exceeding 5%: small yellow croaker, hairtail, anchovy, and Bombay duck. The optimal temperature range for all species was 12–15 °C. Small yellow croaker and hairtail consistently had a suitable temperature range, with the small yellow croaker also displaying a distinct sub-optimal range. Anchovy and Bombay ducks had similar temperature ranges, with anchovy favoring slightly higher temperatures. Salinity suitability followed a similar trend, concentrated between 33 and 34‰. Small yellow croaker and hairtail had overlapping salinity ranges, while anchovy and Bombay duck had similar ranges, with the former group favoring higher salinity. Water depth suitability varied significantly among the four species, with preferences ordered from deep to shallow as follows: hairtail > small yellow croaker > anchovy > Bombay duck.

#### 3.2.2. Seasonal Differences in the Environmental Suitability Selection for Each Fish Species

**Figure 4:**
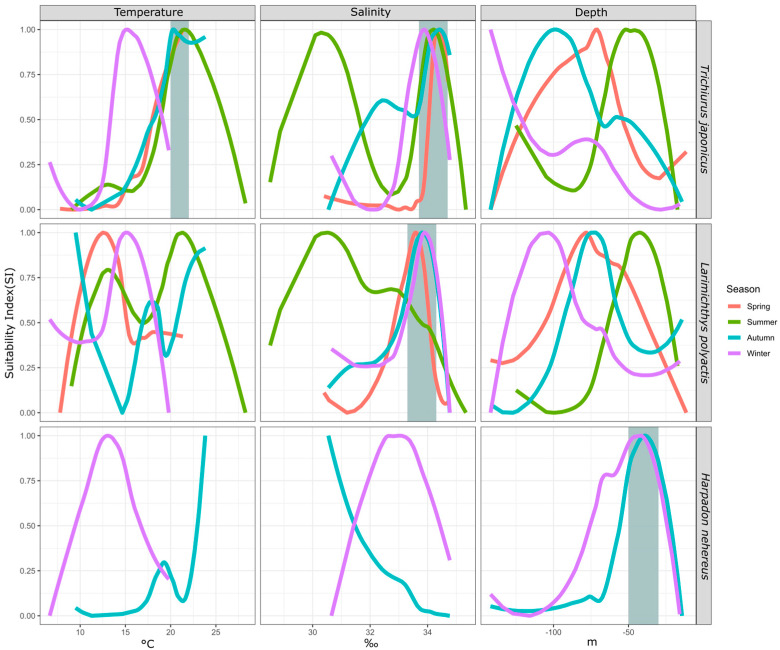
Seasonal difference of environmental SI of major fish species. Note: The gray area indicates the range where the preferred environmental conditions of different species are relatively consistent.

By comparing the suitability of the same fish species for environmental factors across seasons, we can observe changes in their preferences due to seasonal variations. This analysis helps identify the most consistently selected environmental factors, highlighting their significance. This study analyzed three fish species—hairtail (across four seasons), small yellow croaker (across four seasons), and Bombay duck (across two seasons)—over multiple seasons. The results are shown in Figure 4.

For hairtail, the suitable temperature range is 20–22 °C in most seasons, except in winter when it decreases to 15 °C. The suitable salinity range is 34–35‰ across all seasons, with an additional range of 30–31‰ in summer and a secondary range of 32–33‰ in autumn. The preferred water depth is deepest in winter and shallowest in summer, with intermediate preferences in autumn and spring.

For small yellow croaker, the suitable temperature is highest in summer and lowest in autumn, with intermediate preferences in winter and spring. The suitable salinity is 33–34‰ in all seasons, except summer, when it prefers a different range, along with a secondary range close to the primary one. The preferred water depth is deepest in winter and shallowest in summer, with similar depths preferred in spring and autumn.

For Bombay duck, which has higher biomass in autumn and winter, the preferred water depth remains similar in both seasons. However, temperature and salinity preferences show significant differences, with higher temperatures favored in autumn and higher salinity preferred in winter.

### 3.3. Distribution of HSI

**Figure 5:**
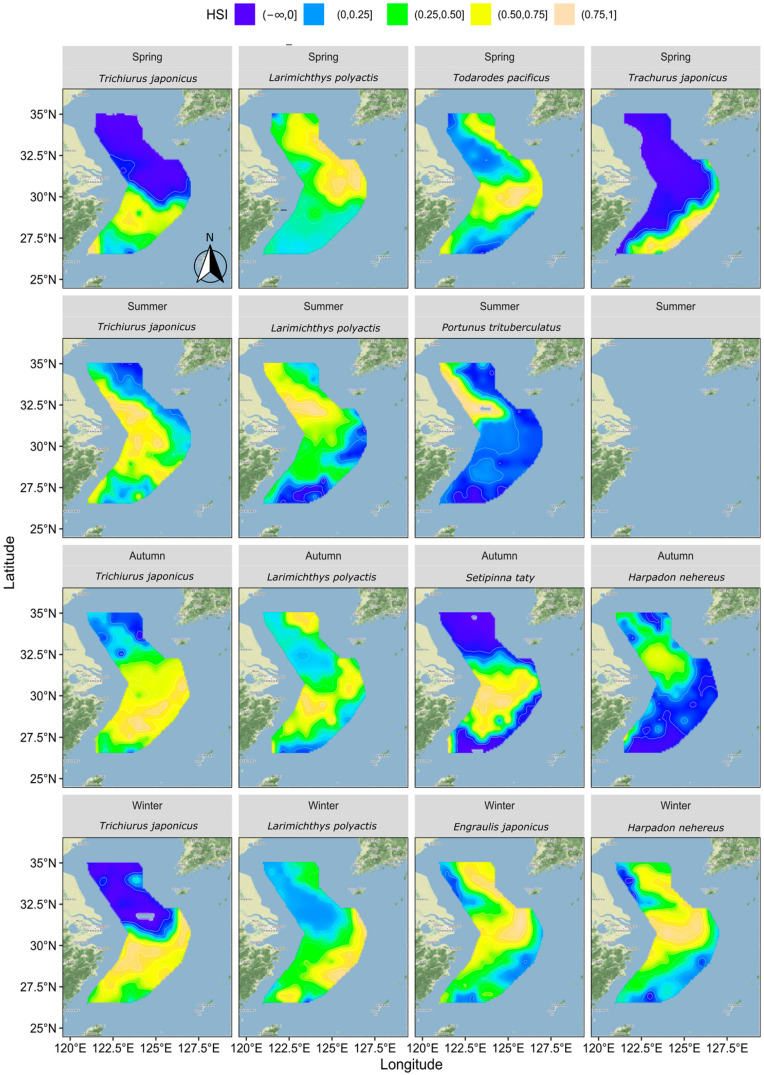
Distribution of HSI for each fish species by season.

Figure 5 illustrates the suitable habitats for each key fish species across four seasons based on their distribution patterns and relationships with hydrological factors.

In spring, hairtail’s suitable habitat was primarily located in the southern waters, while the northern waters were generally unsuitable. The suitable habitat for small yellow croaker was mainly found in the outer northern waters, with lower suitability observed in other areas. Pacific squid’s suitable habitat was concentrated in the central and southern waters, as well as in the outer northern waters, with lower suitability elsewhere. For Japanese jack mackerel, the suitable habitat was mainly in the outer southern waters, with other areas deemed unsuitable.

In summer, hairtail’s suitable habitats extended from south to north along nearshore waters, expanding around the Yangtze River Estuary towards the outer sea. The suitable habitats for small yellow croakers exhibited a similar trend to hairtail but with a broader extension into the outer sea in the northern waters. The suitable habitats for swimming crabs were mainly located in the northern nearshore waters, with some expansion towards the outer sea near the Yangtze River Estuary.

In autumn, compared to summer, hairtail’s suitable habitat disappeared from the northern coastal areas and covered almost all waters south of the Yangtze River Estuary. The suitable habitat for small yellow croaker shifted, with the northern nearshore areas becoming less suitable and the outer sea becoming more suitable. The southern waters also became more suitable for small yellow croaker, both nearshore and in the outer sea. The suitable habitat for Bombay duck was limited, primarily around the Yangtze River Estuary and the northern nearshore area, with other areas deemed unsuitable. Half-fin anchovy’s suitable habitat was concentrated in the middle waters from nearshore to outer sea south of the Yangtze River Estuary, avoiding other regions.

In winter, hairtail’s suitable habitat continued the autumn trend, contracting further south, leaving the northern areas unsuitable. The suitable habitat for small yellow croaker also followed the autumn trend, extending to cover almost all surveyed waters in the south. The suitable habitat for Bombay duck expanded significantly, stretching along the coast to the southernmost end, with a considerable expansion towards the outer sea in the middle and northern regions. The suitable habitat for anchovy resembled that of Bombay duck, with its range extending further towards the outer sea in the south.

### 3.4. Optimal Suitable Habitats Distribution Relationships

**Figure 6:**
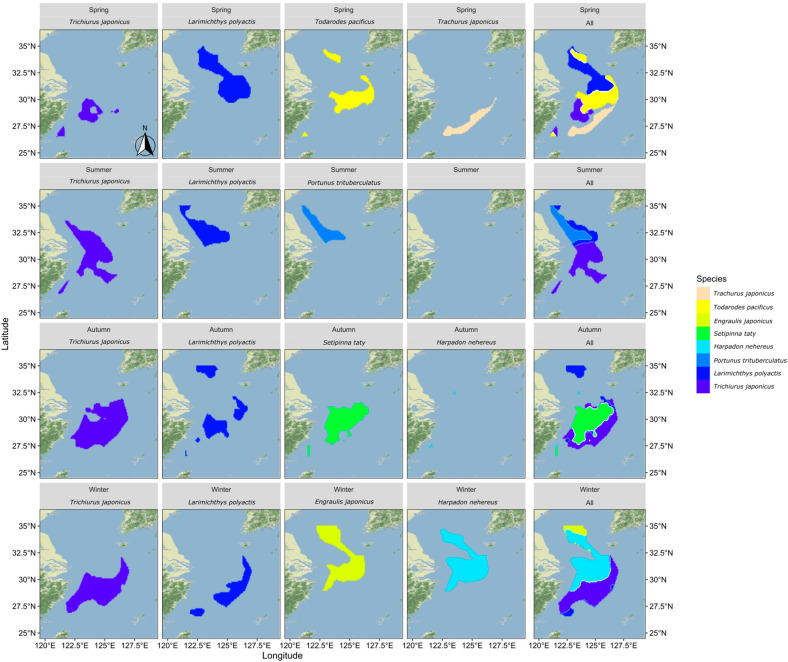
The distribution pattern of optimal suitable habitat for each fish species by season.

**Figure 7:**
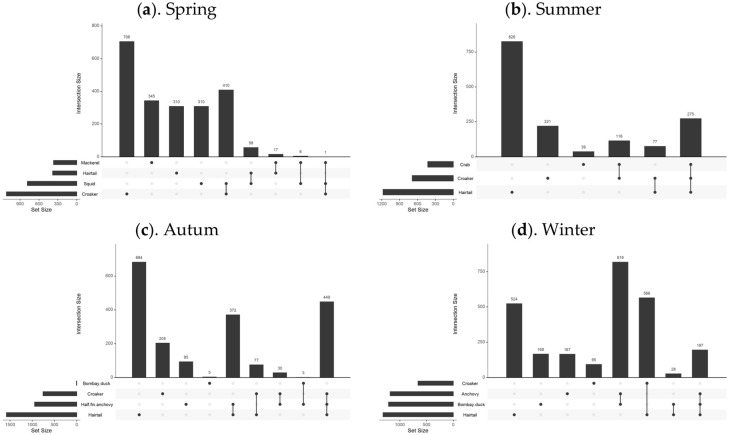
Overlapping relationship of optimally suitable habitats for fish species in different seasons. Note: This figure includes four sub-figures, with labels (**a**–**d**) in the top left corner of each sub-figure, representing the analysis results for the four seasons: spring, summer, autumn, and winter, respectively.

To examine the relationship between the distribution of suitable habitats for dominant fish species across seasons, this study extracted habitats with SI values greater than 0.7 for comparison and overlay, as shown in Figure 6. Upset diagrams were used to illustrate the spatial distribution relationships of different fish species, with the analysis results shown in Figure 7.

In spring, there was minimal overlap between the suitable habitats of the fish species, except for some partial overlap between the most suitable habitats of small yellow croaker and Pacific squid. In summer, the northern coastal area emerged as the most suitable habitat for three predominant species: hairtail, small yellow croaker, and swimming crab. Small yellow croaker and swimming crab were both found in the northern coastal area near the Yangtze River Estuary, with small yellow croaker occupying a wider range. Hairtail’s range extended beyond the first two species, with over half of its distribution located in the southern waters of the Yangtze River Estuary. In autumn, hairtail and half-fin anchovy mainly inhabited the southern waters of the Yangtze River Estuary, with hairtail occupying a broader range. Small yellow croaker’s distribution was dispersed across both the northern and southern regions, with two distinct areas in the south: nearshore and offshore. Bombay duck had a relatively small range, situated in the northern waters outside the Yangtze River Estuary. In winter, the most suitable habitats for hairtail and small yellow croaker showed substantial overlap in the southern offshore waters, while the overlap between anchovy and Bombay duck was more extensive. There was also some overlap between these two groups in the waters outside the Yangtze River Estuary.

### 3.5. Comparison of Overlap Indices by Season

Using to the calculation method described in the methodology section, the spatial overlap indices of species were determined for each season (as shown in Table 2). The results indicate that the overlap index is lowest in spring, displaying negative values. In contrast, the overlap index increases and becomes positive in summer. Furthermore, the overlap index continues to rise in autumn compared to summer, reaching its highest value in winter.

## 4. Discussion

### 4.1. Identification of Key Environmental Factors of the Species’ Habitats

Based on the study results, key environmental factors influencing the spatial distribution of each fish species were identified for each season (refer to Figure 2). The findings indicate that hairtail and small yellow croaker are consistently dominant across all four seasons, while Bombay duck is dominant in only two seasons. Hairtail distribution is primarily influenced by temperature throughout all four seasons. In contrast, small yellow croaker displays varying important environmental factors: temperature in spring and water depth in winter, with no clear factors in summer and autumn. The key environmental factors influencing Bombay duck differ between autumn and winter. Water temperature plays a significant role in shaping hairtail distribution, establishing it as a key factor in defining the ecological niche of this species. However, neither the small yellow croaker nor the Bombay duck exhibited a clear key environmental factor among the three studied, and no single factor remained consistently important across all four seasons.

After analyzing the SI of environmental factors for various species across different seasons (as shown in Figure 4), we observed that the optimal water temperature for hairtail typically falls within the range of 20–23 °C, except during winter when it drops to approximately 15 °C. The optimal salinity range remains consistent across all four seasons, between 33.5 and 34.5‰, with additional suitable ranges of 30–30.5‰ in summer and 32–32.5‰ in autumn. However, the optimal water depth for hairtail varies significantly across seasons. For small yellow croaker, the optimal temperature range varies notably between seasons, while their preferred depth range only slightly overlaps in spring and autumn but differs markedly in other seasons. The optimal salinity range, between 33.5 and 34‰, remains mostly consistent across seasons, except in spring, where it shows a marked difference. For Bombay duck, the optimal temperature and salinity ranges vary across seasons, while their preferred depth remains stable near 50 m. When an organism consistently prefers an environmental factor within a specific range across all seasons, that factor is likely key to its survival and distribution. Based on the above analysis, we conclude that temperature and salinity are the key factors for hairtail, salinity is crucial for small yellow croaker, and water depth is the key factor for Bombay duck.

Using hairtail as an example, we compared our findings with relevant research results from the historical literature. Liu [36] employed frequency analysis to examine the primary water temperature distribution range of hairtail, which aligns closely with our results. Liu’s study indicated that the main water temperature distribution for hairtail across different seasons is 17.95–20.53 °C in spring, 17.48–24.40 °C in summer, 18.11–22.34 °C in autumn, and 14.11–17.90 °C in winter. These values are similar to our findings, which show that the optimal water temperature for hairtail generally falls within 20–23 °C, except during winter when it is approximately 15 ℃. Furthermore, Lin′s research revealed that the salinity range for hairtail in the wintering grounds near Jeju Island is 33.0–34.5‰ [37], nearly identical to our observed range of 33.5–34.5‰. These comparisons demonstrate that the environmental distribution characteristics identified through our SI analysis are reliable, confirming the accuracy of our results.

Ecologists have long been intrigued by the study of key environmental factors that influence biological organisms [38,39]. Numerous studies select a range of environmental factors to examine correlations between fluctuations in the biomass of target organisms and environmental conditions, typically employing statistical models to quantify these correlations, and infer the significance of the environmental factors. For instance, Diankha et al. utilized GAM models to explore the relationship between the recruitment of two sardine species and marine conditions, finding that the pivotal environmental variables influencing the two species differed [40]. The initial approach to defining key environmental factors in this study aligns with the aforementioned method. However, this research employed the BRT model to determine the relative significance of each factor. Despite employing different statistical methodologies, the results were comparable, highlighting the critical role of environmental factors in influencing target organisms. This research adopted a two-step analysis approach. First, it identified important factors for each season and then compared these factors across seasons to determine those that consistently influenced the species, identifying them as key drivers. This approach is more rigorous and presents a greater challenge in identifying key factors. In this study, sea temperature emerged as the only key environmental factor for hairtail, while other fish species did not exhibit a clear key factor under the prevailing environmental conditions. The second method employed is to identify key factors that maintain a high degree of consistency in the optimal range of environmental conditions across all seasons. This approach was grounded in the actual preferences of the organisms, reflecting their unique affinity for specific ranges of environmental factors, which can also be interpreted as the organisms′ fidelity to these particular conditions [41]. This method offers a more flexible screening process, successfully identifying key factors for all three fish species and encompassing the results derived from the first method.

Due to certain uncertainties, the hydrological data for waters south of the 28-degree latitude during the spring and autumn seasons were not obtained from direct measurements; instead, they were substituted with data generated from environmental models [20]. These waters comprise a relatively small portion of the total survey area, accounting for less than one-sixth [42], thereby having a minimal impact on the overall trends observed. Moreover, these southern waters do not constitute the primary distribution area for fishery resources in the East China Sea and, therefore, do not significantly influence the overall distribution pattern of key fishery resources in the region. According to the optimal habitat analysis in this study, aside from hairtail and Japanese jack mackerel, which partially overlap with the most suitable waters in this area, the optimal ranges for other fish species do not intersect with this region. In summary, while the environmental data for waters south of the 28-degree latitude during spring and autumn are modeled rather than directly measured, this does not affect the primary findings and conclusions of the study. Although modeled data might be perceived as less reliable, advancements in environmental data modeling technology are improving the accuracy of predicted environmental data, leading to its broader application [43]. The inclusion of modeled data enhances the comprehensiveness of this study without significantly altering the primary findings and conclusions.

### 4.2. Overlapping Ecological Niche Relationships between Species

The alignment of species with specific environmental conditions defines their unique survival niches [1], which correspond to their roles and positions within the biological community or ecosystem [3]. In this study, various SIs for environmental factors were derived for species within the same season (as depicted in Figure 3). This approach enables the examination of the alignment between species and their environments. The findings reveal varying degrees of overlap or divergence among species across different seasons in relation to specific environmental factors. The greater the overlap between organisms, the higher the convergence of their ecological niches and the greater the likelihood of interaction which may manifest as competition or predator-prey relationships [44].

In August, the three species—hairtail, small yellow croaker, and swimming crab—exhibit a relatively high degree of overlap across various environmental factors. Apart from secondary peaks in water temperature and salinity, all environmental factors present a consistent optimal range. Regarding the spatial distribution of optimal habitats during summer (as shown in Figure 6), a significant overlap occurs near the Jiangsu province shoreline, encompassing shared water areas for the three species. This corresponds with the higher number of shared spatial units among these species, as depicted in the Venn diagram of optimal habitats (see Figure 7b). The ecological niches of the three species, which dominate the biomass in summer, are relatively similar, likely due to common factors driving these species to select similar environments. The overlapping environmental conditions are characterized by higher water temperatures, lower salinity, and shallow water depths (see Figure 3), distinctly different from other seasons. In August, most fish species have completed their breeding and spawning seasons and have transitioned into the feeding migration phase of their life cycle. This water area has a unique geographical environment and is known as a renowned sandbar fishing ground, serving as a spawning ground, nursery area, and crucial feeding ground. Historical surveys [18] confirm that this region is also the feeding ground for the three aforementioned species. Their shared feeding behavior likely contributes to the overlap in ecological spaces. While the overlap of optimal habitats among the three species may primarily result from shared feeding preferences, there is also a simultaneous predator-prey relationship to consider. For instance, small yellow croaker and swimming crabs serve as prey for hairtail [45,46]. A potential causal relationship likely exists among the three species. Specifically, during summer, the waters near Jiangsu in the East China Sea serve as primary feeding grounds for small yellow croaker and swimming crabs, which are prey for hairtail. This dynamic attracts hairtail to this fishing area. Therefore, the distribution patterns of small yellow croakers and swimming crabs may be the cause, with hairtail being drawn to this area for feeding as the resulting effect.

Except for summer, the degree of overlap among various environmental factors for different species is also relatively high in autumn. Although there are four dominant species in autumn, the optimal distribution range of Bombay duck is quite limited, allowing it to be excluded from the analysis of the most suitable habitats in this study. This narrows the focus to the remaining three species: hairtail, half-fin anchovy, and small yellow croaker. As shown in the Venn diagram (see Figure 7c), the shared overlapping space of these three species is the largest among all four seasons, suggesting common factors driving them to occupy similar ecological spaces in autumn. Our study defines November as late autumn, transitioning into early winter. As the water temperature gradually decreases, the fish′s foraging migration period essentially ends, and the overwintering migration begins. The historical literature confirms that the overwintering grounds of the hairtail, half-fin anchovy, and small yellow croaker coincide with the overlap of the areas of highest habitat suitability identified in this study.

High spatial overlap between paired species is observed between small yellow croaker and Pacific squid in spring, hairtail and half-fin anchovy in autumn, and between the Bombay duck and anchovy, as well as hairtail and small yellow croaker in winter. The coexistence of these species within the same water regions may be attributed to the synchronization of their life cycles with the seasons. For instance, breeding behavior in spring (May) and overwintering behavior in autumn (November) and winter (January) can lead to such overlaps [19]. This coexistence may also be influenced by predator-prey relationships between species. For example, a known predation relationship exists between hairtail and small yellow croaker in winter, and the literature suggests that the hairtail may continue feeding even during the overwintering period [18]. Hairtail also preys on half-fin anchovy, exhibiting a predation relationship in autumn [19,47,48]. The nature of interspecific relationships between the remaining paired species requires further analysis in future research.

### 4.3. A Comprehensive Inter-Seasonal Comparison of Overlapping Ecological Niche Relationships among Species

Across all seasons, except for spring, hairtail exhibits the largest optimal habitat area and also has the most extensive non-overlapping area with other dominant species. The other fish species have relatively smaller non-overlapping optimal habitat areas, generally less than a third of that of the hairtail. However, the scenario in spring is different, with small yellow croaker having the largest optimal habitat area, while the other species exhibit optimal habitat areas approximately half the size of the small yellow croaker′s. Examining the seasonal variations reveals distinct differences in the most suitable environmental factors for the four species during spring (as depicted in Figure 3). In contrast, during other seasons, whether considering multiple species or pairs of species, the optimal environmental ranges tend to be more consistent. Additionally, in spring, apart from one pair of species with a substantial overlapping area, the remaining three species exhibit minimal overlap, and other pairs also have relatively smaller overlapping areas. These findings suggest that the ecological niches of these four species are relatively independent during spring.

The overlap indices calculated for each season (as shown in Table 2) align with the previous analysis. Specifically, the results indicate that among the four seasons, the spatial overlap of dominant species is lowest in spring. As the seasons progress, the overlap indices of species gradually increase, peaking in winter. Revisiting the Venn diagrams of species′ spatial relationships across the four seasons (see Figure 7), it is evident that, except for winter, the area with the highest count in other seasons is predominantly occupied by a single species, significantly exceeding the area count of overlapping pairs or multiple species. However, in winter, the area count of two pairs of overlapping species surpasses that of the single species with the highest count. This observation clarifies the calculated results of the comprehensive overlap index for each season. From a biological perspective, the relatively high overlap indices in summer and autumn are primarily due to the larger overlapping areas of multiple species, corresponding to seasonal migration cycles that bring multiple species into environmentally suitable waters. In summer (August), the primary activity is feeding migration, while in autumn (November), the activity shifts mainly to overwintering migration. The significant contribution to the winter overlap index stems from the extensive spatial overlap of two pairs of species, corresponding to the overwintering migration behavior of species. During winter, lower water temperatures generally inhibit predation, resulting in a relatively harmonious coexistence and extensive spatial overlap [19]. In contrast, spring, following the long, cold winter, marks a period of warming temperatures and the revival of life. The intensification of predation and competition among species during this season leads to maximized separation resulting in the lowest overlap observed index in spring [19].

## 5. Conclusions

This study employed two distinct methodologies to identify the key environmental factors influencing the spatial distribution patterns of key fishery resources in the East China Sea. The first method, which focused on factors significant throughout the year, identified water temperature as the critical factor for hairtail. The second method, which considered factors with consistent optimal ranges across seasons, identified additional key factors: water temperature and salinity for hairtail, salinity for small yellow croaker, and water depth for Bombay duck.

The research also explored the ecological niche overlap among different species and seasons. The findings indicated that the main spatial habitat overlap occurs between paired species, likely driven by predator-prey interactions. During summer and autumn, multiple species exhibited increased overlap, primarily influenced by synchronized life cycles. For instance, the physiological requirements for feeding after spawning in summer are aggregated hairtail, small yellow croaker, and swimming crab in the nearshore waters of Jiangsu, which are rich in food resources. In autumn, the physiological demand for overwintering migration brought hairtail, half-fin anchovy, and small yellow croaker together in the waters off central Zhejiang, where water temperatures are relatively high.

An overlap index formula was utilized to quantify species overlap across seasons, showing an increase from spring to winter. This increase reflects a shift in convergent habitat preferences among fish species. The peak overlap occurred in winter, driven by overwintering behavior, reduced food competition, and enhanced coexistence potential. Conversely, the lowest overlap was observed in spring as overwintering concluded and predation and competition intensified.

These insights are valuable for understanding species interactions and effectively managing fishery resources. By identifying key environmental factors and examining ecological niche overlap, this study provides a foundation for developing strategies to sustainably manage fish populations in the East China Sea.

This study utilized the HSI model and two comparative methods to identify key factors influencing fish habitat distribution. By analyzing spatial overlap relationships, we identified both pairwise and multiple overlapping species relationships and observed a gradual increase in the overlap index from spring to winter. The innovation of this study lies in the development of a method based on the SI to identify key factors, the combination of intuitive visualization with overlap calculations to comprehensively analyze spatial overlap relationships, and the introduction of a spatial overlap index for a thorough analysis of overlap degrees. Based on our findings, we recommend that future research further investigate and validate the methods for identifying key factors, explore the driving forces behind species spatial overlap, and examine the underlying causes of variations in the overlap index.

## Figures and Tables

**Table 1:** Major fishery resource species by season.

Season	Species	Biomass (g)	Proportion	Cumulative Proportion
Spring	Hairtail (*Trichiurus japonicus*)	1,620,338	0.36	0.36
	Small Yellow Croaker (*Larimichthys polyactis*)	566,553	0.13	0.49
	Pacific Squid (*Todarodes pacificus*)	297,404	0.07	0.56
	Japanese Jack Mackerel (*Trachurus japonicus*)	290,772	0.07	0.62
Summer	Hairtail	6,074,370	0.41	0.41
	Small Yellow Croaker	3,494,318	0.24	0.65
	Swimming Crab (*Portunus trituberculatus*)	907,367	0.06	0.71
Autumn	Hairtail	893,098	0.23	0.23
	Small yellow Croaker	412,755	0.11	0.33
	Half-fin Anchovy (*Setipinna taty*)	321,371	0.08	0.41
	Bombay Duck (*Harpadon nehereus*)	314,403	0.08	0.50
Winter	Small Yellow Croaker	699,973	0.23	0.23
	Hairtail	356,924	0.12	0.34
	Anchovy (*Engraulis japonicus*)	301,428	0.10	0.44
	Bombay Duck	228,832	0.07	0.52

Note: Biomass—Total survey weight; Proportion—Weight proportion; Cumulative Proportion—Cumulative weight proportion of sorted fish species.

**Table 2:** Calculation results of comprehensive overlap index for each season.

Season	Count Area	Overlap Index
Spring	2163	−0.1583
Summer	1554	0.1287
Autumn	1920	0.4612
Winter	2564	0.5187

## Data Availability

The data presented in this study are available upon request from the corresponding author. The data are not publicly available due to classification as confidential by a government agency, given an association with national security issues.
